# High Rate of Deformed Larvae among Gynogenetic Brown Trout (*Salmo trutta* m.* fario*) Doubled Haploids

**DOI:** 10.1155/2017/2975187

**Published:** 2017-04-09

**Authors:** Krzysztof Jagiełło, Tomasz Zalewski, Stefan Dobosz, Oliwia Michalik, Konrad Ocalewicz

**Affiliations:** ^1^Department of Marine Biology and Ecology, Institute of Oceanography, University of Gdansk, Av. M. Piłsudskiego 46, 81-378 Gdynia, Poland; ^2^Department of Salmonid Research, Inland Fisheries Institute in Olsztyn, Rutki, 83-330 Żukowo, Poland; ^3^Department of Molecular Evolution, University of Gdansk, Ul. Wita Stwosza 59, 80-308 Gdansk, Poland

## Abstract

Mitotic gynogenesis results in the production of fully homozygous individuals in a single generation. Since inbred fish were found to exhibit an increased frequency of body deformations that may affect their survival, the main focus of this research was to evaluate the ratio of individuals with spinal deformities among gynogenetic doubled haploids (DHs) brown trout as compared to nonmanipulated heterozygous individuals. Gynogenetic development was induced by the activation of brown trout eggs by UV-irradiated homologous and heterologous (rainbow trout) spermatozoa. The subsequent exposure of the activated eggs to the high hydrostatic pressure disturbed the first cleavage in gynogenetic zygotes and enabled duplication of the maternal haploid set of chromosomes. The survival rate was significantly higher among gynogenetic brown trout hatched from eggs activated with the homologous UV-irradiated spermatozoa when compared to DHs hatched from eggs activated by the heterologous spermatozoa. More than 35% of the gynogenetic larvae exhibited body deformities, mostly lordosis and scoliosis. The percentage of malformed brown trout from the control group did not exceed 15%. The increased number of deformed larvae among DHs brown trout suggested rather a genetic background of the disease related to the fish spine deformities; however, both genetic and environmental factors were discussed as a cause of such conditions in fish.

## 1. Introduction

Gynogenesis is a form of reproduction in which spermatozoa activate eggs to develop in the absence of paternal chromosomes. Under natural conditions, gynogenesis is observed in some species of fish, amphibians, and reptiles where females from the unisex complexes produce unreduced eggs that are activated by sperm of the related species [1]. However, the gynogenetic development may be induced intentionally. In the artificial gynogenesis, eggs are activated by spermatozoa with the UV-inactivated nuclear genome. The subsequent exposure of the activated eggs to the sublethal temperature or high hydrostatic pressure enables the recovery of the diploid state in the zygotes by inhibition of the second polar body release or suppression of the first mitotic cleavage and production of heterozygous gynogenotes and homozygous gynogenetic doubled haploids (DHs), respectively [2].

Fully homozygous mitotic gynogenotes have been applied in the fish breeding programs, studies concerning the role of recessive alleles during the fish ontogeny, and genome sequencing and gene mapping research [3–5]. Moreover, eggs coming from the gynogenetic DH females may be used for another round of gynogenesis to produce clonal fish [3]. Unfortunately, the potential application of DHs in the aquaculture is limited by a rather low survival rate of mitotic gynogenotes [3, 6, 7].

In general, the low survival rate of DHs results from the expression of recessive traits. Susceptibility to the spinal deformities may be one of the traits reducing the survival of the fish. A high rate of individuals with external malformations, including spine curves observed among farmed fish from inbred strains [8, 9], suggests a genetic component in at least some of the body deformations. Indeed, a genetic basis of scoliosis and kyphosis has recently been described in the model fish species [10, 11]. Larvae with spinal deformities show increased susceptibility to stress and infections [12], impaired swimming abilities [13], and problems with food acquisition [14, 15], which consequently result in their lower growth rate [15, 16] and higher mortality [16, 17]. Spinal deformities in the market-size fish, observed in both marine and freshwater species, cause losses in profits as malformed fish have a lower commercial value and are usually removed from the production or sold at a lower price [18]. Since gynogenetic DHs are inbred and fully homozygous fish, it can be assumed that their high mortality rate partly results from body malformations. Thus, the main objective of the present research was to evaluate whether the rate of individuals with spinal deformities is higher among DH individuals when compared to the noninbred fish. To achieve this objective, we induced gynogenetic development in the nondomesticated brown trout (*Salmo trutta* m.* fario*) using irradiated homologous and heterologous sperm. Survival rates of gynogenotes and normal brown trout were monitored till the swim-up stage. Dead larvae were collected consecutively, individuals with body malformations were counted, and types of deformations were classified based on the body shape and morphology.

## 2. Materials and Methods

### 2.1. Gamete Collection and Induction of the Gynogenetic Development

This study was approved by the Local Committee on the Ethics of Animal Experiments in Gdansk, Poland (number 28/2015). Gamete donors came from broodstocks of the brown trout (*Salmo trutta* m.* fario*) and rainbow trout* (Oncorhynchus mykiss)* kept in the Department of Salmonid Research, Inland Fisheries Institute in Olsztyn, Rutki, Poland. Eggs (*n* = c. 2150) from one brown trout female (BT♀) were stripped, collected in the plastic bowl, and kept in 10°C pending activation. Spermatozoa from one brown trout male (BT**♂**) and one rainbow trout male (RT**♂**) were collected to separate plastic containers. The motility of the collected sperm was checked under a microscope.

### 2.2. Sperm Inactivation by UV Irradiation

Before inactivation by UV irradiation, 0.375 mL of sperm was diluted in 15 mL of the artificial seminal plasma (ASP) (40x) [19]. A 50 mL glass beaker (50 mm diameter) with 15.375 mL of diluted sperm (depth of diluted sperm: 7.8 mm) was placed on a magnetic stirrer and exposed to the UV-C light source for 11 minutes. The distance between the surface of the magnetic stirrer and the UV lamp was 20 cm and the UV intensity was 2075 *μ*W/cm^2^. During the irradiation, diluted sperm was mixed with the magnetic stirrer (1400 G). Irradiated sperm was used for egg activation immediately after the UV exposure.

### 2.3. Egg Activation and Diploidization

Eggs were divided into three batches: two batches contained about 900 eggs in each and one batch contained about 300 eggs. Next, 7.5 mL portions of the diluted and irradiated sperm from brown trout and rainbow trout were used to activate eggs from the first two batches, separately. The remaining eggs were inseminated with normal, nonirradiated brown trout spermatozoa to form the control group (C_BT_) for gynogenetic variants of the experiment. Immediately after addition of the milt, the sperm activation medium (154 mM NaCl, 20 mM Tris, 30 mM glycine, 1 mM CaCl_2_, and pH 9.0) [20] was poured over the batches of gametes, swirled, and left for 3 min. After 3 minutes, activated eggs were thoroughly washed with hatchery water. About 100 eggs activated with irradiated brown trout and rainbow trout were then placed into separate baskets of the egg incubator and form haploid gynogenetic groups, H_BT×BT_ and H_BT×RT_, respectively. The remaining eggs were kept in the water bath adjusted to 10°C for 450 minutes. Then, to double the haploid sets of the maternal chromosomes, eggs from both batches were exposed to high hydrostatic pressure shock (10000 psi), which lasted 3 minutes [21]. Eggs activated with the irradiated brown trout and rainbow trout spermatozoa and subjected to the high pressure shock were named DH_BT×BT_ and DH_BT×RT_, respectively, and placed in separate baskets of the egg incubator. Eggs from the control group and eggs exposed to the high pressure shock were incubated in three replicates at 6–8°C under routine conditions used at the Department of Salmonid Research, Rutki.

### 2.4. Survival and Morphological Development

Measurements of the survival rates were performed at the eyed stage (224 degree-days after insemination), on the day of hatching (441 degree-days after insemination), and at the swim-up stage (597 degree-days after insemination) (Table 1). Dead larvae from the gynogenetic DH_BT×BT_, DH_BT×RT_, and control C_BT_ groups (*n* = 205, *n* = 193, and *n* = 66, resp.) were consecutively collected within four weeks after hatching and placed in absolute ethanol. Fish morphology was then evaluated by analyzing the visible skeletal anomalies including scoliosis, kyphosis, lordosis, and c-shaped and spiraled larvae according to [22, 23] (Table 2).

### 2.5. Molecular Verification of Gynogenesis

DNA was extracted from the fin tissue of parental individuals, dead control, and gynogenetic brown trout larvae (20 from each group) using Genomic Mini AX Tissue (A&A Biotechnology). Homozygosity of the diploid gynogenetic individuals was analyzed with polymorphic microsatellite markers. Microsatellite loci* str543INRA* [24],* str60INRA* [25], and* T3-13* [26] were used for the identification of the parental and gynogenetic genotype. The genetic sex of the gynogenetic offspring was evaluated using the Y-chromosome-related DNA markers* (sdY-Fw)* [27]. PCR reactions were conducted using the Eppendorf Mastercycler Personal thermocycler and the reaction mixture 2xPCR Master Mix (A&A Biotechnology). The reaction conditions for amplification were* str543 INRA*: initial denaturation at 94°C for 4 min, 30 cycles of 94°C for 30 s, 54°C for 30 s, and 72°C for 30 s, and final elongation at 72°C for 10 min;* str60INRA* and* T3-13*: initial denaturation at 94°C for 4 min, 30 cycles of 94°C for 30 s, 61°C for 30 s, and 72°C for 30 s, and final elongation at 72°C for 10 min;* sdY-Fw*: initial denaturation at 95°C for 3 min, 35 cycles of 95°C for 30 s, 60°C for 30 s, and 72°C for 30 s, and final elongation at 72°C for 4 min. PCR products were separated on 1.5% agarose gel (Sigma), stained with ethidium bromide (0.05 mg/mL), and visualized under a UV transilluminator, Vilber Lourmat ECX-20.M. Photos were taken with the Canon PowerShot G16 digital camera.

### 2.6. Statistical Analysis

For the statistical analysis, nonparametric tests were used. The significance of differences between the survival rates of the larvae from the experimental variants was examined by the Kruskal-Wallis test. All calculations were done using Statistica software version 10.1 (StatSoft). A value of *p* < 0.05 was considered statistically significant. All values in the text were expressed as averages ± standard deviations (SD).

## 3. Results

### 3.1. Survival

The survival rate of the brown trout from the control group was above 90% till the swim-up stage (Table 1). In contrast, none of the haploid gynogenetic brown trout embryos survived up to the hatching stage. At the eyed stage, gynogenetic individuals from DH_BT×BT_ group exhibited a significantly higher survival rate than DHs developing in eggs activated by irradiated rainbow trout (and DH_BT×RT_) (Table 1). Nonetheless, in both intra- and interspecies variants of the gynogenesis, DH brown trout larvae hatched and survived till the swim-up stage (Table 1). After hatching, the mortality rate of gynogenotes developing in eggs activated by the irradiated rainbow trout spermatozoa was significantly higher (*p* < 0.05) when compared to gynogenotes induced by UV-inactivated homologous sperm.

### 3.2. Malformations

**Figure 1:**
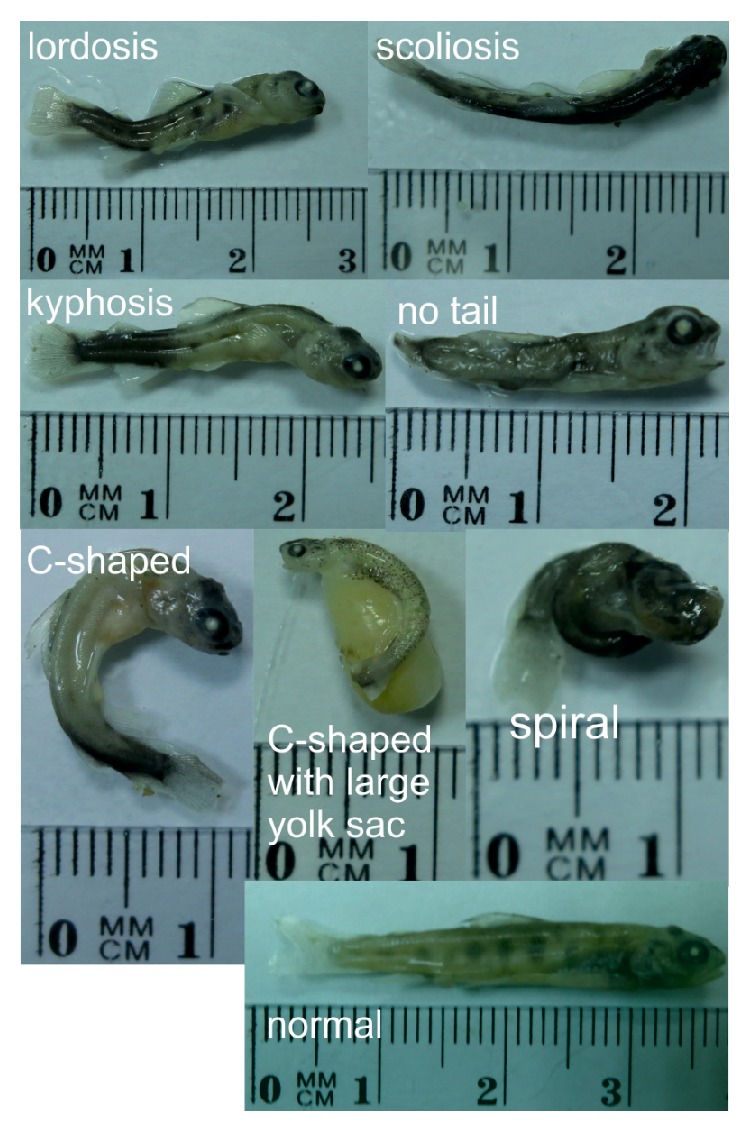
Body deformities among gynogenetic brown trout* (Salmo trutta)* doubled haploids.

Malformation rates among larvae hatched from eggs activated with irradiated brown trout and rainbow trout sperm equaled 35.1% (*n* = 72) and 36.8% (*n* = 71), respectively. The ratio of deformed larvae (*n* = 9, 13.6%) was about three times lower for brown trout from the control group (Table 2). The most common abnormalities were scoliosis and lordosis (Figure 1), whereas larvae with kyphosis and c-shaped and spiral larvae (Figure 1) were less frequently observed. One larva showed no properly developed tail (Figure 1, Table 2).

### 3.3. Homozygosity and Genetic Sex of the Doubled Haploid Brown Trout

Microsatellite polymorphism analysis showed that the female from which eggs were obtained was heterozygous in all the analyzed loci (*543 INRA*,* 60 INRA*, and* T3-13*) (Table 3). Gynogenetic DH larvae showed only one of the alleles present in the female profile, which proves that diploid gynogenetic DH larvae were homozygous in the tested loci (Table 3, Supplementary Figure 1; see Supplementary Material available online at https://doi.org/10.1155/2017/2975187).

Analysis of the* sdY* marker confirmed that only parental males exhibited Y-chromosome-related DNA sequences (PCR product of approx. 400 bp size length). The maternal female and all gynogenetic offspring did not show this marker (Supplementary Figure 2).

## 4. Discussion

Artificially induced mitotic gynogenesis results in the production of fully homozygous fish in a single generation, which makes the mitotic gynogenesis approach less time consuming and less expensive for the production of inbred fish than the traditional inbreeding program. Unfortunately, the high application value of DH individuals is still limited by their low survival rate. DH brown trout hatched from eggs activated by the UV-inactivated homologous sperm exhibited a significantly higher survival rate than those hatched from eggs inseminated with the irradiated heterologous sperm (Table 1). Such phenomenon may be explained by the effect of the paternal factor(s), namely, remnants of chromosomes from the irradiated spermatozoa. Hybrids of brown and rainbow trout are not viable, likely due to the conflict between the egg cytoplasm and the sperm nucleus and the mismatch between chromosomes of the two species [21]. Thus, if a radiation dose applied to spermatozoa was too low to inactivate the rainbow trout nuclear genome entirely, residues of the irradiated chromosomes may provoke a strong reaction of the brown trout egg cytoplasm and genome, leading to increased mortality among the gynogenotes. Such scenario seems to be likely as fragments of UV irradiated paternal chromosomes were observed in gynogenetic specimens [21, 28].

More than thirty percent of the dead brown trout gynogenetic DH larvae showed spinal deformities, with lordosis and scoliosis being the most frequently observed malformations (Figure 1). Vertebral deformities may reduce fish production due to the limited survival of the malformed individuals fish [16, 17]. In fact, brown trout DHs with spinal deformities died within the first weeks after hatching.

The malformation rate in DHs was almost three times higher than the ratio of deformed nonmanipulated brown trout larvae from the control group (Table 2). Although spinal deformities observed in cultured fishes might be explained by environmental factors, including nutritional imbalances, hydrodynamic conditions, and water pollution, the large increase in the number of deformed larvae among DHs raised under identical conditions as the control brown trout suggested also a genetic background of the problem. Our results are paralleled with those obtained in inbred stocks of rainbow trout [8], tilapia* (Oreochromis aureus)* [29], and zebrafish* (Danio rerio)* [30], where the increase of homozygosity was followed by the increased rate of larvae with vertebral deformities including lordosis, scoliosis, and kyphosis. In contrast, larval spinal deformities in chinook salmon* (Oncorhynchus tshawytscha)* were found to be a nonadditive genetic effect and rather result from interactions between parental genomes [31]. However, the knowledge about the genetic background of the spinal deformities in fish is still limited. A genetic component contributing to the spinal deformities in the grass carp* (Ctenopharyngodon idella)* has been proposed [32]. In zebrafish, one of the collagen types (XXVI) has been found to be crucial for the notochord morphogenesis and skeletogenesis. Knockdown of genes encoding collagen resulted in the spinal bone curvature and scoliosis [10], whereas, in guppy* (Poecilia reticulata)*, QTL controlling susceptibility to the spinal curvature has been described. Moreover, this QTL was found to act in a recessive manner [33].

Relatively recently, an overexpression of the* lbx* gene in zebrafish has been found to start a gene cascade leading to scoliosis [11]. Moreover, it has been reported that mutated* lbx* genes may provoke spine deformations during early fish ontogeny as well as during adolescence when females are more susceptible to the disease [11]. The described results suggest that individuals with scoliosis among DH fish may appear also later, after the larval stage of development, which in turn would explain the observations of fish with spinal deformities among one-year-old DH salmonids [34, 35].

On the other hand, some of the spinal deformities might be side effects of the physical treatments applied to the duplicate haploid genome in the gynogenesis process. Crucian carp (*Carassius auratus* Linnaeus 1758) eggs subjected to the hydrostatic pressure shock exhibited impaired embryonic development, including, for example, a delay of epiboly and suppression of the dorsoventral differentiation [36]. Physical shock is also applied to newly fertilized eggs to produce polyploid fishes. Triploid rainbow trout and Atlantic salmon* (Salmo salar)* usually show higher incidences of deformities than diploids [37–39], but it is difficult to evaluate which part of the malformation results from the triploidy itself and the physical treatment. Moreover, studies performed on triploid rainbow trout showed that temperature shock induced a higher rate of deformities than hydrostatic pressure shock [40].

To conclude, the comparison of the body shape among heterozygous control brown trout and homozygous doubled haploid specimens exhibited the threefold increase in the spinal deformities in the DH stock. This suggests that at least part of the vertebral disease has a genetic etiology. Spinal deformities are responsible for some losses in the fish production; however, incidences of scoliosis, lordosis, and kyphosis in fish make them promising animal models in the studies concerning vertebral pathologies in humans.

## Supplementary Material

Supplementary Figure 1: Results of microsatellite DNA analysis (60 INRA, 543 INRA and T3-13 loci) of the brown trout (Salmo trutta) parental individuals and their mitotic gynogenetic offspring. M: DNA ladder, ♂, ♀ – gamete donors, numbered lanes: gynogenetic brown trout DHs, K-: PCR negative control.
Supplementary Figure 2: Examples of the selected brown trout (Salmo trutta) individual genotypes provided in the course of the duplex reaction in the presence of sdY and 18s rDNA primers. Lane 0: DNA ladder, lane 1: brown trout male, lane 2: brown trout female, lanes 3-7: gynogenetic brown trout DHs, lane 8: PCR negative control.

## Figures and Tables

**Table 1:** Survival (%  ± SD) of the normal (C) and gynogenetic doubled haploid (DH) brown trout *(Salmo trutta)* (BT) produced with the use of UV-inactivated homologous (BT) and rainbow trout *(Oncorhynchus mykiss)* (RT) sperm.

Experimental group	Eyed stage	Hatching stage	Swim-up stage
DH_BT×RT_	44.7 ± 4.97	23.7 ± 4.24	22.3 ± 3.50
DH_BT×BT_	66.5 ± 5.56	42.1 ± 3.17	41.4 ± 2.85
C_BT_	96.4 ± 2.46	91.9 ± 1.30	90.7 ± 1.90

**Table 2:** Summary of the examination of the body morphology among gynogenetic doubled haploid (DH) and normal (C) brown trout *(Salmo trutta)*.

Body morphology	DH_BT×BT_	DH_BT×RT_	C_BT_
Normal	133	122	57
Lordosis	31	39	6
Scoliosis	33	22	2
C-shaped larvae	2	2	0
Spiral larvae	1	3	0
C-shaped larvae with enlarged yolk sac	2	1	0
Multiple scoliosis	0	3	0
Kyphosis	2	0	1
Kyphosis with enlarged yolk sac	0	1	0
Without a tail	1	0	0

*Rate of deformed larvae*	*35.1%*	*36.8%*	*13.6%*

**Table 3:** Results of microsatellite genotyping of the maternal brown trout specimen and its gynogenetic progenies.

Locus	Maternal genotype	Gynogenetic progeny genotype	The number of gynogenetic progeny
543 INRA	120/160	120/120	5
		120/160	0
		160/160	15

60 INRA	90/110	90/90	11
		90/110	0
		110/110	9

T3-13	200/235	200/200	12
		200/235	0
		235/235	8
