# Supplementary figures and images for: High Rate of Deformed Larvae among Gynogenetic Brown Trout (Salmo trutta m. fario) Doubled Haploids
Source: Biomed Res Int. 2017 Apr 9;2017:2975187. doi: 10.1155/2017/2975187 (PMC5401714; doi:10.1155/2017/2975187)

Figure 1

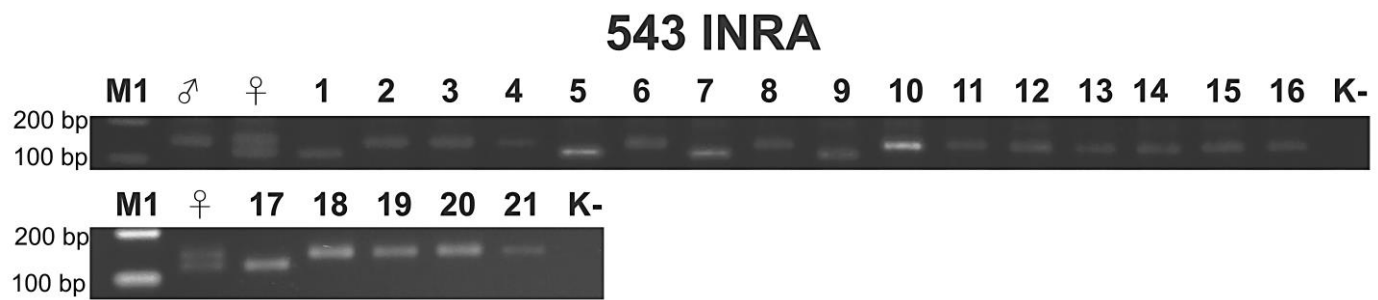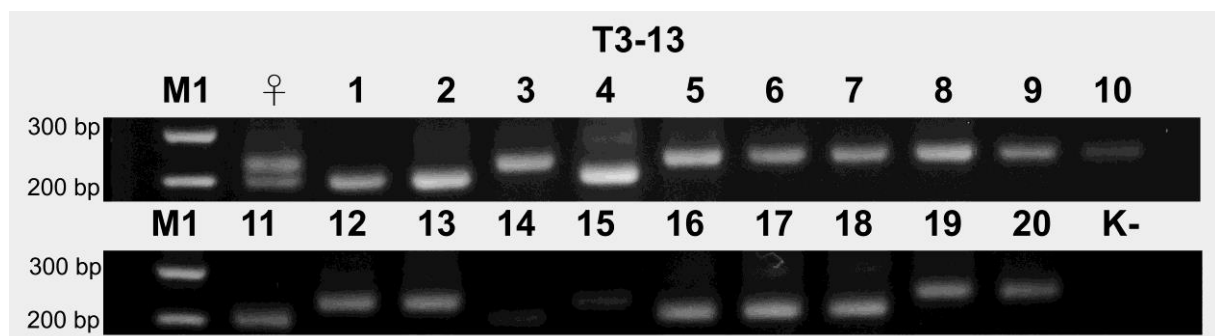

Figure 2

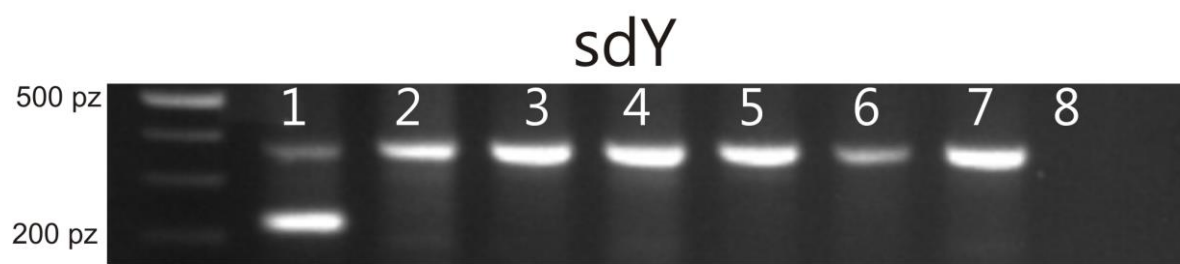

Supplement: Supplementary file 1 — Supplementary Figure 1: Results of microsatellite DNA analysis (60 INRA, 543 INRA and T3-13 loci) of the brown trout (Salmo trutta) parental individuals and their mitotic gynogenetic offspring. M: DNA ladder, ♂, ♀ – gamete donors, numbered lanes: gynogenetic brown trout DHs, K-: PCR negative control. Supplementary Figure 2: Examples of the selected brown trout (Salmo trutta) individual genotypes provided in the course of the duplex reaction in the presence of sdY and 18s rDNA primers. Lane 0: DNA ladder, lane 1: brown trout male, lane 2: brown trout female, lanes 3-7: gynogenetic brown trout DHs, lane 8: PCR negative control. [file 2975187.f1.pdf]
